# Sensor-Fused Nighttime System for Enhanced Pedestrian Detection in ADAS and Autonomous Vehicles

**DOI:** 10.3390/s24144755

**Published:** 2024-07-22

**Authors:** Jungme Park, Bharath Kumar Thota, Karthik Somashekar

**Affiliations:** College of Engineering, Kettering University, Flint, MI 48504, USA; thot3744@kettering.edu (B.K.T.); soma3420@kettering.edu (K.S.)

**Keywords:** ADAS, nighttime object detection, sensor-fusion, image alignment, Deep Neural Network, transfer learning, embedded devices

## Abstract

Ensuring a safe nighttime environmental perception system relies on the early detection of vulnerable road users with minimal delay and high precision. This paper presents a sensor-fused nighttime environmental perception system by integrating data from thermal and RGB cameras. A new alignment algorithm is proposed to fuse the data from the two camera sensors. The proposed alignment procedure is crucial for effective sensor fusion. To develop a robust Deep Neural Network (DNN) system, nighttime thermal and RGB images were collected under various scenarios, creating a labeled dataset of 32,000 image pairs. Three fusion techniques were explored using transfer learning, alongside two single-sensor models using only RGB or thermal data. Five DNN models were developed and evaluated, with experimental results showing superior performance of fused models over non-fusion counterparts. The late-fusion system was selected for its optimal balance of accuracy and response time. For real-time inferencing, the best model was further optimized, achieving 33 fps on the embedded edge computing device, an 83.33% improvement in inference speed over the system without optimization. These findings are valuable for advancing Advanced Driver Assistance Systems (ADASs) and autonomous vehicle technologies, enhancing pedestrian detection during nighttime to improve road safety and reduce accidents.

## 1. Introduction

Environmental perception plays a crucial role in the development of intelligent automotive systems, such as Advanced Driver Assistance Systems (ADASs) and Autonomous Driving (AD). This involves the utilization of Artificial Intelligence (AI) technology to process automotive sensor data, enabling obstacle detection and perception of a vehicle’s surrounding. Since AlexNet [1] won the ImageNet Large Scale Visual Recognition Challenge (ILSVRC) [2] in 2012, various deep Convolutional Neural Network (CNN) algorithms have emerged, such as VGG16/19 [3], InceptionNet [4], ResNet [5], DarkNet [6], etc., each making significant contributions to the field. Due to these innovative CNN algorithms, current environmental perception systems utilizing visual RGB camera sensors have achieved state-of-the-art performance.

The incorporation of recent DNN architectures, like the series of YOLO algorithms [7,8], has enabled these perception systems based on RGB camera sensors to excel in real-time inferencing during the daytime. This is accomplished by effectively utilizing the visual spectrum as input data. Nevertheless, the performance of these systems experiences a significant drop in scenarios with reduced visibility, such as during nighttime or adverse weather conditions like fog, rain, glare from the sun, and other challenging situations. RGB cameras are dependent on external light sources, so in low light conditions, they are unable to take clear pictures of the environment around them. This leads to poor-quality images being fed to the detection algorithm, which in turn leads to improper or missed detections of objects [9].

**Figure 1:**
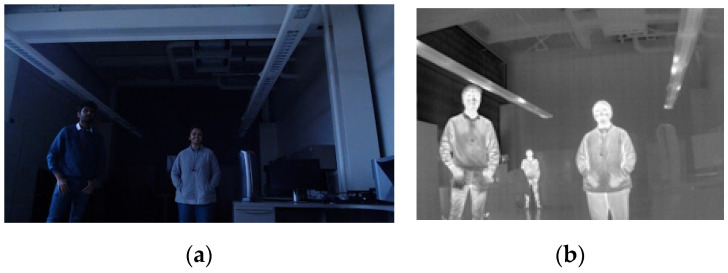
(**a**) A 960 by 540 RGB image in which the third person in the back is not captured by the RGB camera due to low lighting conditions; (**b**) a 640 by 512 IR thermal image of the same scene.

Infrared (IR) thermal imaging serves as an alternative solution to address nighttime perception challenges in AD and ADASs. By detecting heat radiation from objects and capturing variations in heat distributions among different object types, a thermal infrared camera effectively portrays objects with higher heat radiations in a brighter manner. Notably, the IR thermal sensor operates without visible lights. This makes it a reliable choice for nighttime and adverse weather conditions such as fog, rain, overcast, dust storms, and more [9]. Figure 1 displays images captured by both standard RGB and IR cameras under the low light condition at the same time. The initial image in Figure 1a represents an RGB image with a resolution of 960 by 540. Due to the low illumination conditions, the person at the back is not captured in the RGB camera image. On the other hand, the IR thermal image with a resolution of 640 by 512 in Figure 1b shows the person at the back clearly. This example shows thermal imaging remains unaffected by lighting conditions and provides consistent image output regardless of light availability.

An IR camera offers the advantage of identifying objects even in complete darkness, highlighting the potential for developing a detection system primarily based on IR technology. However, IR thermal cameras also have limitations. They rely on detecting infrared radiation emitted by objects, primarily driven by temperature differences. Consequently, when target objects have temperatures similar to their surroundings, IR cameras may produce unclear depictions of their shapes, leading to false positives or misclassifications in an IR camera-only system [10]. Therefore, it is crucial to research integrating information from both IR and RGB cameras to explore the fusion effect of combining RGB and IR thermal imaging. This integration aims to enhance the object detection system for AD and ADASs in low-light conditions, potentially improving accuracy and reliability.

**Figure 2:**
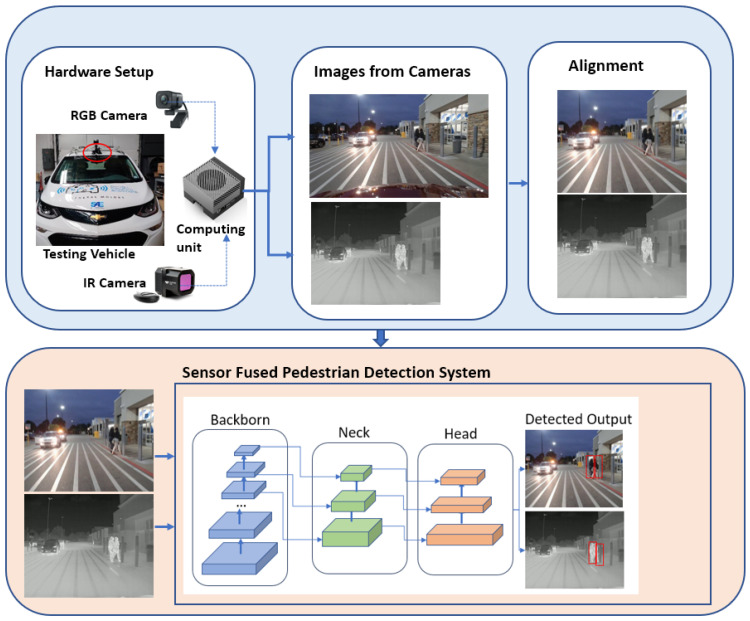
The overall architecture of the proposed nighttime pedestrian detection system: sensors mounted on the vehicle indicated by the red circle, with detection results presented using red bounding boxes.

This paper explores a sensor-fused nighttime environmental perception system by integrating data from IR thermal and RGB cameras with a proposed innovative alignment algorithm and advanced Deep Neural Network (DNN) technologies. The overall system architecture is presented in Figure 2. Initially, the test vehicle is equipped with RGB and IR thermal cameras, and an edge computing device (the NVIDIA Jetson Orin (Nvidia, Santa Clara, CA, USA) [11] is integrated as an embedded computing component. The aligned images from both sensors are then input into the trained sensor-fused DNN system for pedestrian detection during nighttime. After the optimization process, the proposed system, deployed on the in-vehicle computing system, is able to run at 33 frames per second (fps) for real-time inferencing. The major contributions of this paper are as follows: (1) A novel image alignment algorithm is proposed. Given the distinct fields of view (FOVs) and spatial resolutions of the two sensors, a new innovative alignment algorithm is introduced to automatically align the images from both sensors without manually measuring their displacement in the x, y, and z directions, which is prone to errors and cumbersome. (2) To develop a robust nighttime pedestrian detection system, a total of 32,000 new nighttime data samples were collected and labeled. Five DNN models were developed, including three fusion methods and two single-modal systems, using 110,000 data samples and transfer learning. (3) The best-performing DNN model for the nighttime pedestrian detection system was optimized for enhanced performance. Initially, due to the limited computing power of the in-vehicle system, the running time for the best DNN model dropped from 55.65 fps on a Dell laptop (Dell, Round Rock, TX, USA) to 18 fps on the in-vehicle computing unit. However, through optimization, the fused DNN model’s running time improved from 18 fps to 33 fps. This optimization enabled the system to perform real-time inferencing.

This paper is organized as follows. In Section 2, an extensive literature review is conducted on sensor-fused object detection applicable to both daytime and nighttime scenarios. Section 3 outlines the comprehensive methodology for developing a sensor-fused nighttime pedestrian detection system, integrating thermal and RGB cameras. Moving forward, Section 4 delves into a discussion of the experimental results with different sensor fusion methods. The paper concludes with Section 5, summarizing the findings and insights obtained from the study.

## 2. Related Work

Ensuring safe nighttime AD and ADASs relies on the early detection of vulnerable road users and animals like deer, with minimal delay and high precision. Several researchers have delved into various techniques for extracting data from both RGB and thermal cameras. The authors in [12,13] proposed the thermal camera only method. In [12], the authors explored object detection using solely thermal images. They employed a DNN trained on RGB images for object detection in the thermal domain. Their framework utilized a Single Shot Detection (SSD) architecture with MobileNet as its backbone. Although they reported decent detection results, the system’s performance was compromised because it was initially trained on RGB datasets, lacking proper tuning for thermal images. The authors in [13] also proposed a thermal-only object detection system. They conducted experiments by adjusting image quality parameters and observed that the system’s accuracy was contingent upon the quality of the IR thermal images. Additionally, they integrated a low-pass filter to effectively diminish the brightness of high-temperature objects while enhancing their contours. This dual effect significantly reduced noise, resulting in more precise object detection outcomes.

In early fusion, RGB and IR thermal images are spatially aligned and merged at the pixel level to form a single image [10,14,15,16]. Early fusion methods involve combining pixel-level information from images captured by an IR thermal camera and an RGB camera, resulting in a single image that is then processed by an object detector. Zhou et al. [10] proposed a Resnet-based method for feature extraction from thermal images, incorporating a channel attention mechanism to enhance region of interest (ROI), albeit with slow computation. In [14], the focus is on early fusion with the YOLO-RGB-T model, modifying YOLOv4’s input layer to accommodate both RGB and thermal images. This model achieved mAPs of 64.8% in daytime and 60.9% at nighttime, surpassing single-sensor systems. The authors in [15] introduced an early fusion model for pedestrian detection, combining feature maps from color and thermal branches, followed by Network-in-Network (NIN) to reduce dimensionality. Trained on the KAIST multispectral pedestrian dataset, this model exhibited reduced miss rates compared to single-modality models. The authors in [16] evaluated fused RGB–Long Wave IR (LWIR) object detection for air-based platforms, achieving an mAP@50 of 75%, outperforming RGB-only (25%) and LWIR-only (70%) models, particularly in challenging conditions.

The mid-fusion strategy improves the object identification algorithm by extracting and fusing the information from each sensor before feeding the feature data to the detector head. Several studies explore mid-fusion strategies for RGB and thermal data in object detection [17,18,19,20,21,22]. In [20], the authors introduced object detection based on mid fusion using two sensor combinations: (1) a radar with an RGB camera, and (2) an IR thermal camera with an RGB camera. Their study concluded that leveraging complementary sensors enhanced the object detection model’s precision by three times when compared to the no-fusion model. Similarly, ref. [21] proposed a mid-fusion approach with a redesigned version of YOLOv5, featuring a two-stream backbone for low-light object detection. These backbones extracted features from RGB and thermal images, which were fused in a Cross-modality Fusion Transformer (CFT) module to generate enriched features. They reported that their model with the CFT module demonstrated superior performance compared to other experiments conducted. The CFT-based models reported an increase of 6% in mAP over the no-fusion models.

Lastly, the late-fusion method involves making decisions after independent detections on RGB and thermal images. A final detection is determined by combining the confidence scores of the individual detections [23,24,25,26]. In [23], the authors implemented the Retina Net algorithm independently on the RGB and IR images. Then, they applied non-maximum suppression to combine the individual sensor outputs. Sousa et al. [24] used YOLOv5 for object detection, where objects in RGB and thermal images are detected separately. Then, they employed a fully connected multi-layer neural network to combine the outputs from each sensor. In [25], the authors used YOLOv3-tiny for object detection on individual sensors. In addition, they also used connected components in thermal images to leverage detection accuracy. Late fusion was implemented into a single confidence map. Yang et al. in [26] used YOLOv4 for individual sensor object detection. They proposed an Illumination-Aware Network (IAN) to decide which model to trust based on paired RGB/IR images, generating object detection results and confidence weights.

In [27], the authors proposed a method to improve human detection in AD systems by integrating selective thermal imaging data with RGB images. Their approach utilizes the RGB camera for initial object recognition and obstacle detection, with thermal cameras activated selectively to detect pedestrians under conditions such as obscured views or low light. This targeted use of thermal imaging significantly enhances pedestrian recognition accuracy. YOLOv5 was employed to train on a customized dataset of 2156 images for object and lane recognition models, supplemented by the FLIR dataset for pedestrian recognition using thermal cameras. They reported an increase in object recognition accuracy from 40.43% with RGB images alone to 83.91% when incorporating thermal image data. The system was implemented on Nvidia Jetson Nano, achieving a processing speed of 0.75 fps. However, this processing speed of 0.75 fps poses a bottleneck, rendering it unsuitable for real-time inferencing.

Image alignment is a critical procedure for fusing information from different camera sensors. In [28], the authors discussed the calibration and image registration of RGB-D and thermal cameras, including UV cameras. They emphasized a two-point approach to equalize epipolar geometries and employed registration techniques to align the images. This method relies on detecting and matching features between images using common feature descriptors like SIFT, SURF, and BRISK. However, these methods are computationally demanding, making them less suitable for real-time processing applications due to their high computational cost.

Image alignment in the KAIST dataset [29] involves a specialized hardware setup comprising a color camera, a thermal camera, a beam splitter, and a three-axis camera jig. The beam splitter aligns the optical centers of both cameras by transmitting the thermal band and reflecting the visible band. Calibration is performed using stereo camera calibration techniques to eliminate translation between the optical axes of the cameras. A special heated calibration board with holes is used for fine-tuning the alignment. Post-processing corrects color distortions caused by the beam splitter using a reference image of a white plane. In the LLVIP dataset [30], the image alignment process is described through image registration. This method includes manually identifying several corresponding points between the two images, calculating a projection transformation to adjust the infrared image, and then cropping the images to produce accurately aligned pairs.

The KAIST dataset [29] employs a specialized hardware setup with a beam splitter and a special heated calibration board for image alignment, while the LLVIP dataset [30] uses manual identification of common points for image registration. To simplify the image alignment process and avoid additional hardware costs and manual identification of common points, we developed a novel approach using Deep Neural Networks (DNNs). This approach automatically identifies and uses common points generated by bounding boxes of the same objects, without manual measurement and additional hardware, to correct misalignment issues.

## 3. Development of a Sensor-Fused Nighttime Obstacle Detection System

As illustrated in Figure 2, the development of the nighttime object detection system encompasses several steps. These include hardware setup and sensor selection, data collection, image alignment across different sensors, information fusion, training the object detection models, evaluating the developed models, and deploying the system for real-time inference. In this paper, the authors propose a novel method for image alignment from two different sensors, collect and label 32,000 paired data samples from the IR and RGB cameras, and implement three different sensor-fusion methods. The best-performing sensor-fused DNN model was optimized for deployment on the in-vehicle computing unit.

### 3.1. Hardware Setup for a Sensor-Fused Nighttime Obstacle Detection System

**Figure 3:**
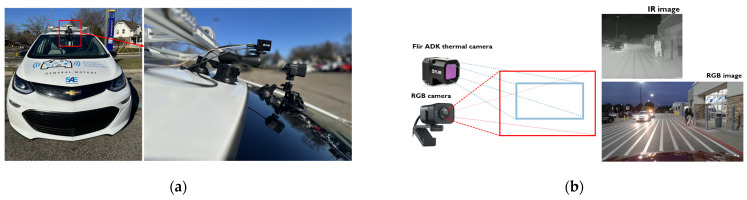
(**a**) Hardware setup on the testing vehicle; (**b**) captured images with different FOVs and resolutions.

As presented in Figure 3a, this research utilizes a multi-sensor configuration integrated into a Chevrolet Bolt platform. The FLIR ADK IR thermal camera [31] serves as a pivotal component, offering high-resolution thermal imaging capabilities at a 640 × 512 resolution with a 50° field of view (FOV) and a rapid capture rate of 60 fps. Complementing this, the Logitech (Logitech, San Jose, CA) Stream Cam [32] is employed to record RGB video at a resolution of 960 × 540 with a 78° FOV, also at 60 fps. The IR thermal camera is mounted on the roof of the car and the RGB camera is located on the windshield of the car near to rear-view mirror. Figure 3b presents the captured images with different FOVs and resolutions. To enable real-time inferencing for object detection under low illumination conditions, the NVIDIA Jetson Orin [11] computing device is utilized as an in-vehicle computing unit, leveraging its robust computational capabilities and 32 GB RAM. Table 1 summarizes the specifications of the in-vehicle computing unit, the NVIDIA Jetson Orin.

### 3.2. Alignment of Two Different Sensor Images

For a sensor-fusion system incorporating RGB and IR thermal cameras, image alignment is essential to integrate data from two different sensors. The necessity for image alignment emerges from inherent disparities in sensor placements, orientations, and perspectives, potentially leading to misalignments in captured images. As presented in Figure 3b, images are captured from two sensors with different resolutions and FOVs. The Logitech RGB camera image has a 960 × 540 resolution with a 78° FOV, and the FLIR ADK thermal camera (Teledyne FLIR, Wilsonville, OR, USA) has a 640 × 512 resolution with a 50° FOV. Because the two cameras also have different fields of view (FOVs), a parallax effect is observed between images of the same scene captured by the two cameras. The change in the FOV causes a parallax phenomenon, which displaces an object differently due to the varying FOV. If images from two different sensors are not aligned properly, they can result in the erroneous fusion of features, complicating the fusion algorithm’s ability to accurately combine information from various sources.

**Figure 4:**
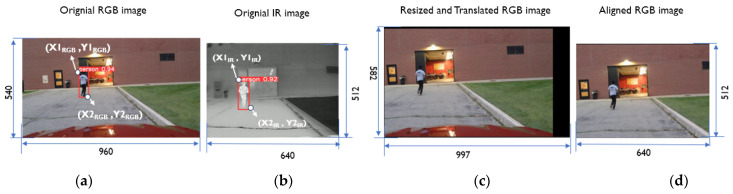
The proposed image alignment method involves: (**a**) an original RGB image with dimensions of 960 × 540 pixels and a field of view (FOV) of 78°; (**b**) an original IR image sized at 640 × 512 pixels with a FOV of 50°; (**c**) a resized and translated RGB image; and (**d**) the aligned RGB image corresponding to the IR image.

To resolve the parallax issue, the authors proposed a new image alignment algorithm that determines the necessary parameters to align images from different camera sensors. The proposed alignment algorithm generates resizing and translating parameters for alignment by comparing the location information of the same object on the images from two different cameras. Since RGB and IR thermal images capture the same two-dimensional scene, the factors contributing to misalignment are positional and size differences. Given that the IR image has a lower FOV (a 50° FOV) compared to the RGB image’s FOV (a 78° FOV), the RGB image will be aligned with respect to the IR image. The procedures for the proposed alignment algorithm are described in the following steps:

Step (1) Capture paired images containing a single object (e.g., a pedestrian) using two cameras mounted on the test vehicle, as illustrated in Figure 3a. The authors utilized 20 paired images.
Step (2) For each pair of RGB and IR images:
  (i) Detect the object using the existing DNN model [33]: The DNN-based object detection algorithm is separately applied to both RGB and IR images, resulting in bounding box coordinates (depicted in Figure 4a and Figure 4b, respectively). In Figure 4a, the RGB image detection is represented by coordinates (*X1_RGB_*, *Y1_RGB_*) for the top-left and (*X2_RGB_*, *Y2_RGB_*) for the bottom-right. Similarly, the IR image detection in Figure 4b uses coordinates (*X1_IR_*, *Y1_IR_*) and (*X2_IR_*, *Y2_IR_*).
  (ii) Calculate the resizing factor: To quantify size differences between images from different sensors, resizing factors in the x and y directions are computed using Equations (1) and (2):
    *RFactor_X* = (*X2_IR_* −* X1_IR_*)/(*X2_RGB_* − *X1_RGB_*). (1)
    *RFactor_Y* = (*Y2_IR_* − *Y1_IR_*)/(*Y2_RGB_* − *Y1_RGB_*). (2)
    Here, *RFactor_X* represents the ratio of the IR image bounding box width to the RGB image bounding box width, and *RFactor_Y* calculates the ratio of the IR image bounding box height to the RGB image bounding box height.
  (iii) Calculate the translation factor: Positional differences between RGB and IR images arise from field of view (FOV) variations. Translation adjusts the RGB image coordinate system to align with the IR image coordinate system. Translation parameters in the x and y directions are determined using Equations (3) and (4):
    *TranFactor_X* = (*X1_IR_* − *X1_RGB_*) × *RFactor_X*. (3)
    *TranFactor_Y* = (*Y1_IR_* − *Y1_RGB_*) × *RFactor_Y*. (4)
  (iv) Record these four parameters: resizing factors and translation factors in the x and y directions.
Step (3) For each parameter, calculate the average value using the data generated in Step 2.

Table 2 presents the parameters derived from 20 pairs of images using the proposed alignment algorithm. Using these calculated resizing and translation parameters, the RGB image is resized and translated accordingly. Figure 4c displays the output post-translation operation. Following translation, the RGB image is cropped to 640 by 512 pixels, starting from the top-left coordinate (1, 1) to (640, 512), matching the size of the IR image as shown in Figure 4d. The proposed alignment algorithm requires a single run during camera calibration. Once alignment parameters, resizing factors, and translation factors are computed using this method, they enable real-time alignment of RGB and IR images in subsequent operations. The algorithm is efficient and robust, facilitating the development of sensor-fusion algorithms across different camera sensors.

**Figure 5:**
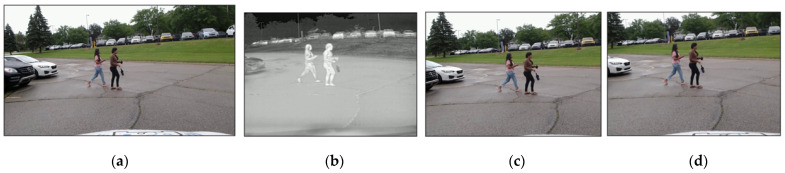
The image alignment results comparison: (**a**) an original RGB image with dimensions of 960 × 540 pixels and a field of view (FOV) of 78°; (**b**) an original IR image sized at 640 × 512 pixels with a FOV of 50°; (**c**) the aligned RGB image using the registration method; and (**d**) the aligned RGB image using the proposed method.

Figure 5 displays the image alignment results produced by both the existing registration method and the proposed image alignment method. Figure 5a,b depict the original RGB camera image and its corresponding IR thermal image, respectively. In Figure 5c, the output from the current registration method is shown. A comparison with the corresponding IR thermal image reveals noticeable misalignment, particularly in areas such as trees, cars, and pedestrian locations. In contrast, Figure 5d presents the output from the proposed method, demonstrating accurate alignment with the corresponding IR thermal image.

### 3.3. Publicly Available Dataset and New Data Collection

**Figure 6:**
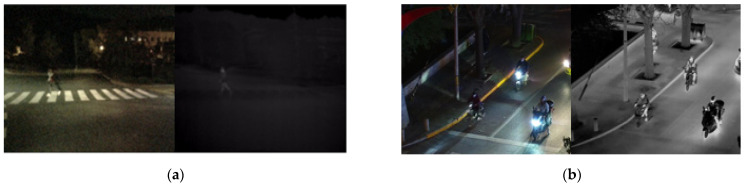
Public datasets for nighttime pedestrian detection: (**a**) an example from the KAIST dataset with poor IR image quality; (**b**) an example from the LLVIP dataset.

To develop the nighttime pedestrian detection system, two publicly available datasets were used: the KAIST dataset [29] and the LLVIP (Low Light Vision Pedestrian) dataset [30]. The KAIST dataset [29], published in 2015, initiated low-light object detection research. This dataset consists of pairs of aligned RGB and thermal images, all with a resolution of 640 × 512 for pedestrian detection. The second dataset is the LLVIP (Low Light Vision Pedestrian) dataset [30], which comprises pairs of RGB and thermal images taken in low-visibility scenes, with all images in the dataset spatially aligned. Example images from these datasets are shown in Figure 6a,b for the KAIST dataset and the LLVIP dataset, respectively. However, these two datasets have several drawbacks. The KAIST dataset suffers from extremely poor IR image quality, as shown in Figure 6a, while the LLVIP dataset consists of images captured by surveillance cameras, which do not align with the viewpoint of the cameras mounted on vehicles.

**Figure 7:**
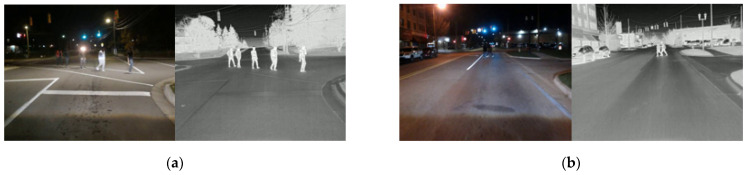
The Kettering dataset collected by the authors: (**a**) Pedestrian crossing; (**b**) Urban driving scenario.

Therefore, the authors decided to collect data that better suited the requirements for the night model development. The authors gathered data across various scenarios, including residential and urban driving, pedestrian crossings, shopping malls, and parking lots, during nighttime and low-light conditions. In total, 55,000 frames of nighttime data were collected, with 32,000 frames containing pedestrians. Sample images collected by the authors are presented in Figure 7.

All collected images were aligned using the proposed alignment algorithm explained in Section 3.2, then labeled using the MATLAB Image Labeler app [34]. Three different datasets, the KAIST, the LLVIP, and the Kettering datasets, were utilized to develop the nighttime pedestrian detection system. The data samples are categorized into three groups for training, validation, and testing of the DNN models. A summary of the entire dataset utilized is provided in Table 3.

### 3.4. Development of the Sensor-Fusion DNN Models

**Figure 8:**
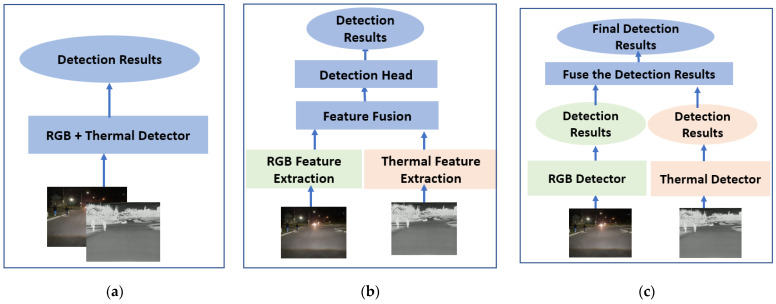
Three different sensor-fusion methods: (**a**) early fusion; (**b**) mid fusion; (**c**) late fusion.

Using a single sensor for object detection can lead to vulnerabilities, as it may fail to provide adequate information in certain scenarios (e.g., obscured vision due to low lighting conditions or fog). Sensor fusion mitigates these risks by providing redundant or complementary data from multiple sensors, making the system more robust and reliable in various environmental conditions. For sensor-fusion systems, how data are integrated from different sensors is critical to the overall system performance. Figure 8 shows three different fusion methods that combine the data differently: early fusion, mid fusion, and late fusion [19].

Early Fusion: As depicted in Figure 8a, the early-fusion method integrates input images from multiple sensors at the beginning of a data processing pipeline, before the DNN model. The objective is to create a unified and comprehensive representation of the scene by leveraging the complementary nature of RGB and thermal information. The IR and RGB images are fused using the weighted sum method [35], which employs a mathematical approach to combine multiple values. Each value is multiplied by a specific weight, reflecting its significance in the overall decision-making process. The following procedures are applied to fuse the images from two sensors:

(i) Each RGB image is aligned using the proposed image alignment algorithm in Section 3.2. The aligned RGB has the same image width, *imgW*, and image height, *imgH*, as the corresponding IR thermal image.
(ii) To generate the fused representation of the scene captured by two different sensors, the proposed weighted sum approach involves adding two weighted pixel values at each location (*x*, *y*) for each color channel.

For every channel *c*, where *c* = 1:3 in an RGB image:

For every pixel location (*x*, *y*):

*Fused_img*(*x*, *y*, *c*) = (*IRimg*(*x*, *y*) × *W*_*IR*_) + (*RGBimg*(*x*, *y*, *c*) × *W_RGB_*) (5)

where *IRimg* is an IR image and *RGBimg* is an RGB image. The ranges of *x* and *y* are defined as *x* = [1: *imgH*], *y* = [1: *imgW*]. The weights, *W_IR_* and *W_RGB_*, are associated with the IR image and the RGB image, respectively, where *W_IR_* + *W_RGB_* = 1. The fused image data, *Fused_img*, will be used to develop the DNN models. In this research, a 60/40 ratio of IR to RGB images is utilized based on experimental findings, where 60% of the total weight is attributed to IR images and 40% to RGB images.

**Figure 9:**
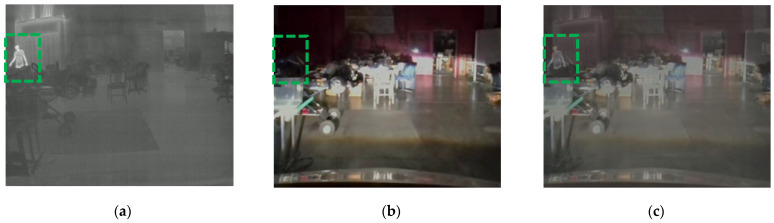
The weighted sum method for early fusion: (**a**) the person in the green box is captured in the IR image even in low lighting conditions; (**b**) the person in the green box is not captured in the RGB image due to low lighting conditions; (**c**) the person in the green box is captured in the weighted sum image by fusing the IR and RGB images.

**Figure 10:**
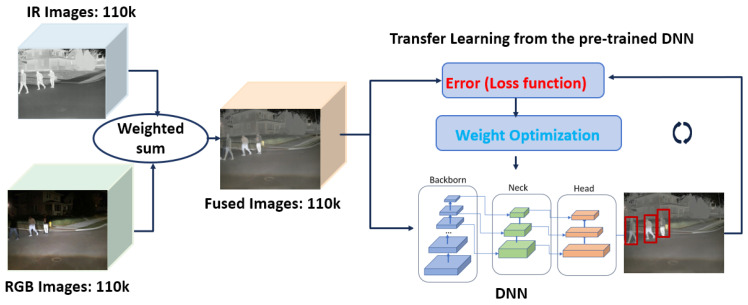
The training of the early-fusion model using transfer learning.

Figure 9 shows an example of information fusion using the weighted sum method with the weights *W_IR_* = 0.6 and *W_RGB_* = 0.4. Figure 9a,b are taken from the IR camera and the RGB camera, respectively. As shown in Figure 9b, the RGB camera image did not capture the details of the person (marked with a green dotted rectangle) under low-light conditions. On the other hand, the IR image in Figure 9a shows the details of the person in the same scene. The fused image using the weighted sum method displays the person in the same scene as presented in Figure 9c. The early-fusion model is trained with 110,000 training samples in Table 3. Rather than training from scratch, the model is developed using the transfer learning method [36] with the pre-trained YOLO v5 [33] model, as shown in Figure 10.

**Figure 11:**
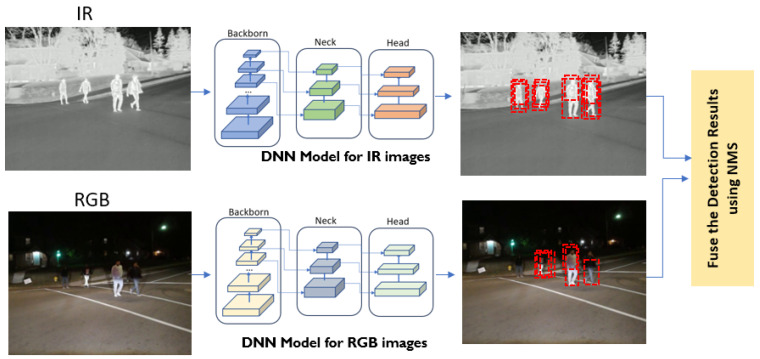
The architecture of the late-fusion model.

Late Fusion: Late fusion is a technique that involves merging detection results after independent detections on RGB and IR thermal images. This approach allows for the utilization of diverse types of data or DNN models, potentially leading to improved performance or robustness compared to using any single modality or model in isolation. Late fusion contrasts with early fusion, where data from different sources are combined before being fed into DNN models. In Figure 11, an overview of the late-fusion method is presented, illustrating how RGB and IR images are separately input into the object detection DNN models. Each detection includes details such as bounding box information and confidence scores for each detected object. The outcomes from each sensor are compared and merged, as shown in Figure 11, using the Non-Maximum Suppression (NMS) algorithm [37].

**Figure 12:**
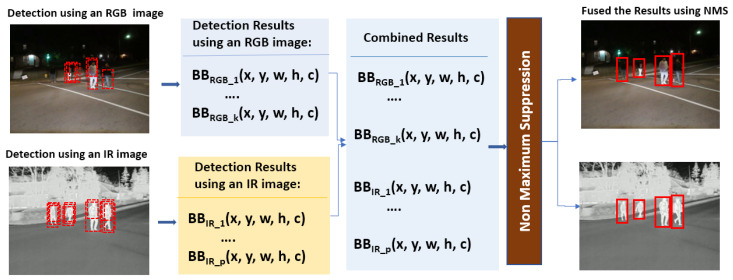
The NMS procedure for late fusion.

The NMS algorithm [37] is a post-processing technique designed to remove redundant detections of the same object within a single DNN model’s output. When an image is input into an object detection model, it identifies objects based on features such as hands, legs, and other body parts. Consequently, the model’s output may include duplicate detections for a single object, as shown in the dotted bounding boxes in Figure 11 and Figure 12. Moreover, the application of NMS can be extended to merge detection results from different DNN models originating from various sources (such as RGB and IR), ensuring that each object is associated with the most accurate bounding box. This process enhances the accuracy and reliability of object detection, as illustrated in Figure 12. The NMS process for the late-fusion system involves the following steps:

Step 1 Merge detection results in the form of a set of bounding boxes along with their associated confidence scores from two different object detection DNN models.
Step 2 Sort the bounding boxes based on their confidence scores in descending order. This ensures that the box with the highest confidence score is considered first.
Step 3 Start with the bounding box that has the highest confidence score, *high_bb,* in the sorted list. This box is considered a potential detection.
Step 4 Iterate over remaining boxes in the sorted list.

For each box, *bb_i*, in the sorted list:

i. Calculate the intersection over union (IoU) with the current bounding box, *bb_i,* and the highest confidence score bounding box, *high_bb*.
  IoU = | *high_bb* ∩ *bb_i* |/| *high_bb* ∪ *bb_i* | (6)
ii. If the IoU is above a certain threshold (0.5 is used), discard the bounding box, *bb_i*, as it significantly overlaps with the currently selected box, *high_bb,* and is likely to represent the same object; otherwise, keep the bounding box.

Steps 3 and 4 are iteratively applied to the next highest confidence score bounding box until no additional bounding boxes remain. Applying NMS eliminates redundant detections, resulting in a cleaner and more accurate set of bounding boxes for object detection tasks in late fusion.

**Figure 13:**
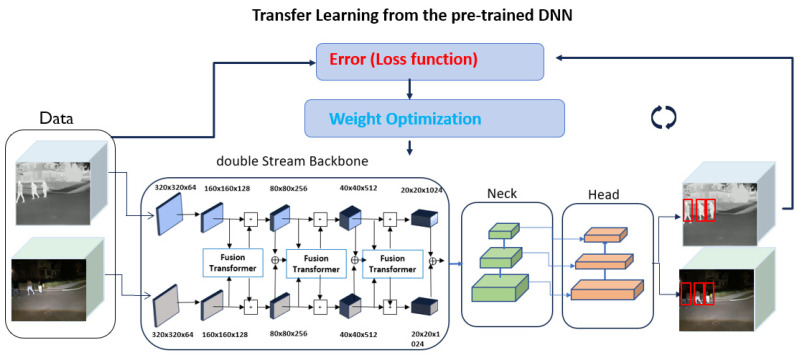
Training of the mid-fusion method using transfer learning.

Mid Fusion: To implement the mid-fusion method using RGB and IR images, the original YOLO v5 algorithm was redesigned with dual-stream backbones, as described in [21] and illustrated in Figure 13. This approach processes RGB and IR thermal images separately: the first stream backbone extracts features from RGB images, while the second backbone extracts features from thermal images. The key component of this architecture is the CFT modules [21], where features from RGB and IR thermal images are integrated. The proposed mid-fusion model is trained using transfer learning with 110,000 data samples, as shown in Table 3. Integrating RGB image features with thermal image features enhances feature richness. These enriched features are then reprocessed through the RGB backbone and, similarly, thermal images are enhanced with RGB features and reprocessed through the thermal backbone. This fusion of features improves detection across multiple scales.

Training for five DNN models, including three fusion models and two single-mode models, was conducted on a Dell Alienware Aurora R8 desktop computer with a 9th Gen Intel Core i7-9700 processor and an NVIDIA GeForce RTX 2080 Ti GPU. Each model was trained with 110,000 paired data samples as detailed in Table 3. For all Deep Neural Network (DNN) models, the authors used a learning rate of 0.001 and the Stochastic Gradient Descent (SGD) optimizer, with a batch size set to 12.

## 4. Experimental Results and Deployment for Real-Time Inferencing

### 4.1. Experimental Results of the Pedestrian Detection System under Low Lighting Conditions

The performance of different DNN models was evaluated using testing samples from three datasets (KAIST, LLVIP, and Kettering), described in Table 3. The performance metrics used in the evaluation include precision, recall, mAP50, F1-Score, and fps (frames per second). Precision measures the proportion of correct positive predictions among all positive predictions, while recall calculates the percentage of correct positive predictions among all positive cases in the data [38]. mAP50 captures the tradeoff between precision and recall at an IoU threshold of 50%. The F1 score, which considers the harmonic mean of precision and recall, offers a comprehensive measure of the balance between the two metrics. The computational time of each model, measured in fps, was obtained using a Dell Alienware m15 R7 laptop computer equipped with an Intel Core i7-12700H and an NVIDIA GeForce RTX 3070 Ti.

The developed DNN models were evaluated using testing datasets from three different databases. The testing data samples from the KAIST dataset have poor-quality IR thermal images because they were generated in 2015. A total of 5000 samples from the KAIST dataset were used to evaluate five different models. Table 4 summarizes the detection results of these 5000 KAIST testing data samples. The experimental results of the KAIST dataset confirm the trend of superior performance among fused models compared to non-fusion counterparts, with mAP50 values of 54.3% for the camera-only model, 62.3% for the thermal-only model, 64.1% for the early-fusion model, 69.4% for the mid-fusion model, and 65.9% for the late-fusion model. The low performance of the DNN models on the KAIST dataset is attributed to the poor quality of IR images, as presented in Figure 6a.

Table 5 summarizes the results of the 2200 LLVIP testing data samples (referenced in Table 3), confirming the trend of superior performance among fused models compared to non-fusion counterparts, with mAP50 values of 75.4% for the camera-only model, 96.1% for the thermal-only model, 96.9% for the early-fusion model, 97.5% for the mid-fusion model, and 97.3% for the late-fusion model. Similarly, Table 6 presents the performance of the five DNN models on 6000 testing samples from the Kettering dataset, with mAP50 values of 72.8% for the camera-only model, 91.2% for the thermal-only model, 92.6% for the early-fusion model, 95.6% for the mid-fusion model, and 95.5% for the late-fusion model. All experimental results underscore the consistent advantage of fused DNN models in achieving a higher mAP50 across test samples in the three different datasets.

To benchmark our test results, we compared them with state-of-the-art algorithms. Specifically, we utilized two DNN models for nighttime pedestrian detection from previous works cited in [21] and [30]. The DNN model in [30] employed a thermal-only strategy and reported a 94.6% mAP50 on the LLVIP dataset. In contrast, our thermal-only model, trained on three different datasets as shown in Table 3, achieved a 96.1% mAP50 on the LLVIP dataset, demonstrating an approximate 1.59% improvement over the DNN model in [30]. Additionally, we compared the DNN model in [21] to our mid-fusion model, which was trained on the three datasets using transfer learning, as shown in Table 3. The mAP50 scores of the DNN model in [21] were 63.5%, 97.2%, and 87.6% on the KAIST, LLVIP, and Kettering testing samples, respectively. On the other hand, our mid-fusion model achieved mAP50 scores of 69.4%, 97.5%, and 95.6% on the KAIST, LLVIP, and Kettering testing samples, respectively. These results indicate improvements of 9.29%, 0.31%, and 9.13% on the KAIST, LLVIP, and Kettering testing samples, respectively.

For processing time, the camera-only model and the thermal-only model achieve fast processing times of 66.67 fps and 67.11 fps, respectively. The early-fusion model operates at 60.25 fps because it fuses data from two different sensors before passing it to the DNN model. In contrast, the mid-fusion and late-fusion models have slower processing times of 45.25 fps and 55.65 fps, respectively. The mid-fusion model exhibits the slowest processing time due to the data processing steps in the backbone, as illustrated in Figure 13.

### 4.2. Deployment of the DNN Model for Real-Time Inferencing

To deploy a sensor-fused pedestrian detection system under low lighting conditions, achieving low response time and high accuracy is critical for automotive applications. Considering these requirements, a late-fusion system is selected for its balance of accuracy and response time. The late-fusion DNN model for the pedestrian detection system under low lighting conditions can run at 18 fps on the NVIDIA Jetson Orin, whose specifications are presented in Table 1. To further improve inference time using the TensorRT engine [39], the DNN model can be optimized through several techniques: (1) Vertical fusion of kernels to perform sequential operations together. (2) Horizontal fusion of layers into a single layer if they share the same input and filter size but have different weights. (3) Elimination of unnecessary layers through model analysis. The steps for optimizing the DNN model are as follows:

1. Convert the trained DNN model into the ONNX format [40].
2. Convert the ONNX model into the TensorRT engine [39]. During this conversion, the network graph is restructured to enhance operational efficiency.

Detailed optimization procedures can be found in [41].

**Figure 14:**
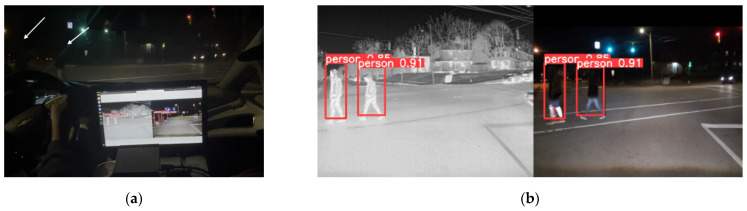
Nighttime testing example 1: (**a**) Due to the low lighting conditions, two pedestrians on the left side (indicated by white arrows) are not visible. (**b**) Pedestrians are correctly detected by the sensor-fused system and marked with red bounding boxes on the screen.

**Figure 15:**
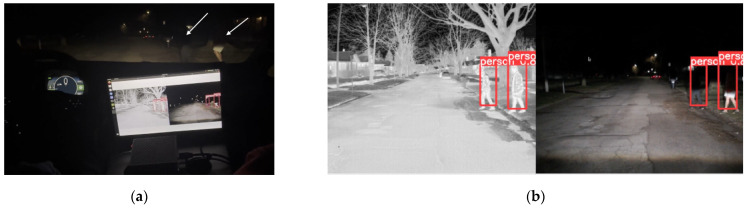
Nighttime testing example 2: (**a**) Due to the low lighting conditions, two pedestrians on the right side (indicated by white arrows) are not visible. (**b**) Pedestrians are correctly detected by the sensor-fused system and marked with red bounding boxes on the screen.

**Figure 16:**
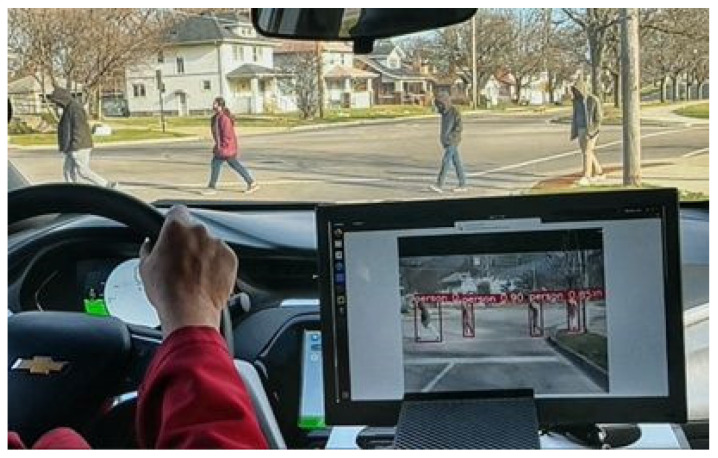
Real-time inference results during the daytime. The proposed system can be run during the daytime and correctly detects pedestrians.

Once the DNN model in PyTorch is converted into the TensorRT engine, it is deployed onto the embedded computing device, NVIDIA Jetson Orin. The optimized DNN model runs at 33 fps, improving the processing time by approximately 83.33% from the 18 fps of the PyTorch version. Figure 14 and Figure 15 present examples of the detection results from the optimized DNN model during nighttime. In Figure 14a and Figure 15a, pedestrians are crossing at the intersection area. Due to the low lighting conditions, the pedestrians are not clearly visible, as indicated by the white arrows in Figure 14a and Figure 15a. The optimized sensor-fusion system correctly detects the pedestrians, as shown in Figure 14b and Figure 15b. Additionally, the proposed system is also applicable during the daytime, as presented in Figure 16.

## 5. Conclusions

This paper explores a sensor-fused nighttime environmental perception system by integrating data from IR thermal and RGB cameras. To fuse the data from these two different sensors, the authors propose a new alignment algorithm. The proposed alignment algorithm resizes and translates RGB camera images to match the width and height of IR thermal images. Given that the images from these two sensors have distinct FOVs and spatial resolutions, the new alignment algorithm aligns the images for subsequent sensor-fusion processing. This alignment process is crucial as it constitutes a pivotal step in fusing information from different sensors. The aligned images from both sensors are then input into the trained sensor-fused DNN system for pedestrian detection.

To develop a DNN-based pedestrian detection system for low lighting conditions, the authors collected nighttime images in various scenarios and labeled a set of 32,000 IR and RGB image pairs. To explore different fusion methods, three fusion techniques (early, mid, and late) were developed using transfer learning technology. Additionally, two single-sensor models were developed using either RGB camera data only or IR thermal camera data only. In total, five different DNN models were developed and evaluated on the testing data samples from three different datasets.

The experimental results confirmed the trend of superior performance among fused models compared to their non-fusion counterparts. For example, the five DNN models achieved mAP50 values of 72.8% for the RGB camera-only model, 91.2% for the IR thermal-only model, 92.6% for early fusion, 95.6% for mid fusion, and 95.5% for late fusion on the Kettering testing data samples. For processing time, the camera-only model and the thermal-only model achieved fast processing times of 66.67 fps and 67.11 fps, respectively. The early-fusion, mid-fusion, and late-fusion models operated at 60.25 fps, 45.25 fps, and 55.65 fps, respectively. Considering the low response time and high accuracy requirements, a late-fusion system was selected for its balance of accuracy and response time.

When the late-fusion model was deployed on the in-vehicle computing unit, NVIDIA Jetson Orin, the processing time dropped from 55.65 fps to 18 fps. For real-time inferencing, the selected model was further optimized, achieving 33 fps on the embedded edge computing device, representing an 83.33% improvement in inference speed over the system without optimization. The findings in this paper are valuable for advancing ADAS and AD technologies in low lighting conditions, enhancing pedestrian detection at nighttime to improve road safety and reduce accidents.

## Figures and Tables

**Table 1:** NVIDIA Jetson Orin specification.

Parameter	Specification
RAM	32 GB
GPU/CPU	56 Tensor Cores
GPU max frequency	930 MHz
CPU max frequency	2.2 GHz

**Table 2:** Parameters for the proposed alignment algorithm.

Parameter	Specification
Resizing factor in the x direction	1.04
Resizing factor in the y direction	1.08
Translation factor in the x direction	16.50
Translation factor in the y direction	12.95

**Table 3:** Datasets used to develop the nighttime pedestrian detection system.

	KAIST	LLVIP	Kettering	Total
Training	80,000	10,000	20,000	110,000
Validation	5000	2200	6000	13,200
Testing	5000	2200	6000	13,200

**Table 4:** Evaluation of the DNN models on the KAIST dataset.

DNN Models	Precision/Recall	F1-Score	mAP50	fps
RGB only	55.8/52.8	54.26	54.3	66.67
Thermal only	65.7/57.6	61.4	62.3	67.11
Early Fusion	70.3/63.0	66.5	64.1	60.25
Mid Fusion	75.1/68.3	71.5	69.4	45.25
Late Fusion	75.5/63.6	69.0	65.9	55.65
Model in [21]	66.5/58.7	62.4	63.5	42.12

**Table 5:** Evaluation of the DNN models on the LLVIP dataset.

DNN Models	Precision/Recall	F1-Score	mAP50	fps
RGB only	88.5/70.2	78.3	75.4	66.67
Thermal only	97.0/89.3	93.0	96.1	67.11
Early Fusion	97.3/90.8	93.9	96.9	60.25
Mid Fusion	97.7/91.8	94.7	97.5	45.25
Late Fusion	97.5/91.3	94.3	97.3	55.65
Model in [21]	97.3/91.2	94.2	97.2	42.12

**Table 6:** Evaluation of the DNN models on the Kettering dataset.

DNN Models	Precision/Recall	F1-Score	mAP50	fps
RGB only	87.3/63.4	73.45	72.8	66.67
Thermal only	93.2/88.7	90.9	91.2	67.11
Early Fusion	94.7/90.4	92.5	92.6	60.25
Mid Fusion	96.8/91.6	94.1	95.6	45.25
Late Fusion	97.1/90.8	93.8	95.5	55.65
Model in [21]	96.6/80.2	94.1	87.6	42.12

## Data Availability

The data presented in this study are available on request from the corresponding author.
